# Facts and Fancies

**Published:** 1897-02

**Authors:** 


### FACTS AND FANCIES.

Mr. Benjamin F. Poole, of Rockland, Mass., maintains an equine graveyard for the members of the horse family that die in his possession.

The study of phrenology as applied to the horse is attracting more and more attention as its importance becomes better understood.

"Old Ned," nearly forty-two years of age, once black, now grizzled gray, his head white, still lingers under the kindly care of his owner, Mr. B. F. Crawford, Erie, Pa., who received him while on duty in the rebellion in 1864.

August 12, 1874, Goldsmith Maid, made the first mark of 2.15 as a trotter; in twenty years the number has reached nearly five hundred, all of these performers being recorded in full in the Christmas number of the *Spirit of the Times.*

Seven head of Dorset Horn Sheep have just been purchased by a lady and presented to the Schenley Park Gardens at Pittsburg, Pa.

Kentucky veterinarians are strongly in favor of the movement having for its object the securing of the 1897 meeting of the U. S. V. M. A., at Nashville.

The January number of the *American Veterinary Review* contains a photogravure frontispiece of the newly elected officers of the United States Veterinary Medical Association. It is well prepared, and will be much appreciated by the members of the Association.

Many progressive Western farmers are urging the establishment of a government stud, so that choice blood may be obtained at the smallest cost. They think it would be better to spend the money in this direction than in distributing worthless government seeds, causing so great a scandal and waste of public moneys.

Philadelphia consumed during 1896, 97,000,000 quarts of milk. This was gathered from 3300 farms, averaging 15 head of cattle each. The daily supply represents 47 carloads of 150 cans to a car. But 3 to 5 per cent. of this entire supply was found adulterated by the Board of Health through its inspection department.

One of the most noted ancient racers was Phrenico. He belonged to Hiero, of Syracuse, and won the race in the seventy-third Olympiad.

The remains of a fossil horse, about twenty inches high, have been found in Utah, Wyoming, and other parts of the Rocky Mountain regions.

According to Simmonds, Europe had in 1890, 34,865,000 horses; Asia, 4,443,000; Africa, 721,000; America, 21,920,000, and Australia, 1,520,000.

Incitatus was the horse of Emperor Caligula, and was made priest and consul. He had an ivory manger, and drank wine out of a golden pail.

"Nutwood's" grave will be fittingly marked with a suitable stone.

One of Philadelpha's foremost veterinarians, who some years ago completed a course of human medicine, is now gradually entering upon that branch of practice.

The suggestion of the *Breeders' Gazette* for a thorough veterinary department at the Illinois State University, is meeting with warm approval by agriculturists throughout the State.

